# Clinical, molecular, and immunologic determinants of survival in WHO-defined IDH-wildtype glioblastoma treated with radiotherapy: a large real-world cohort study

**DOI:** 10.1007/s11060-026-05572-w

**Published:** 2026-04-25

**Authors:** Cole Friedes, Melanie Berger, Lauren Linkowski, Casey Hollawell, Harper Hubbeling, Daniel Alexander, Goldie Kurtz, Robert A. Lustig, Jay F. Dorsey, Arati S. Desai, Richard E. Phillips, Steven Brem, Christos Davatzikos, MacLean Nasrallah, Donald M. O’Rourke, Christina Jackson, Nduka M. Amankulor, Michelle Alonso-Basanta, Suyash Mohan, Stephen J. Bagley, Emily S. Lebow

**Affiliations:** 1 Department of Radiation Oncology, Hospital of the University of Pennsylvania, Philadelphia, PA USA; 2 Department of Neuro-Oncology, Hospital of the University of Pennsylvania, Philadelphia, PA USA; 3 Department of Neurosurgery, Hospital of the University of Pennsylvania, Philadelphia, PA USA; 4 Division of Neuroradiology, Hospital of the University of Pennsylvania, Philadelphia, PA USA; 5 Division of Neuropathology, Hospital of the University of Pennsylvania, Philadelphia, PA USA; 6 3400 Civic Center Boulevard, Philadelphia, PA 19104 USA

**Keywords:** High grade glioma, Glioblastoma, Lymphopenia, Prognostic factors, Molecular GBM

## Abstract

**Introduction:**

Glioblastoma (WHO grade 4), defined by IDH-wildtype status and associated molecular features, carries poor prognosis, and real-world survival models incorporating molecular and immunologic variables remain limited. Severe radiation-induced lymphopenia (sRIL) is a proposed prognostic factor, but its independent effect in molecularly defined glioblastoma has not been established in the modern era.

**Methods:**

We retrospectively identified 832 adults with glioblastoma, defined as WHO 2021 grade 4 IDH-wildtype diffuse glioma treated with maximal safe resection and adjuvant radiotherapy (RT) with or without chemotherapy between 2014 and 2024. A trial-eligible subgroup was defined using Stupp criteria. Clinical, molecular, and hematologic variables were analyzed. sRIL was defined as CTCAE grade ≥ 3 lymphopenia within 4 months of RT. Multivariable Cox models incorporated LASSO for variable selection and spline regression for non-linearity.

**Results:**

Median overall survival (OS) was 13.0 months. On multivariable analysis, sRIL (HR 1.37, 95% CI 1.16–1.63) remained an independent predictor of worse OS after adjustment for MGMT status, age, resection extent, and treatment factors. MGMT methylation predicted benefit from concurrent TMZ (p_interaction_<0.001), and trial-eligible patients had longer OS across MGMT subgroups. Age and post-RT lymphocyte nadir were non-linearly associated with survival. Proton therapy was associated with a favorable but non-significant OS estimate (HR 0.87, *p* = 0.11), with no benefit in the trial-eligible cohort.

**Conclusions:**

In this large, real-world cohort, sRIL remained a strong independent prognostic factor. MGMT methylation predicted TMZ benefit, and trial eligibility conferred favorable outcomes. These findings underscore the prognostic relevance of post-treatment lymphopenia and support prospective evaluation of lymphocyte-sparing treatment strategies.

**Supplementary Information:**

The online version contains supplementary material available at 10.1007/s11060-026-05572-w.

## Brief key points

• Severe post-RT lymphopenia is independently associated with worse survival in IDH-wt glioblastoma.

• MGMT methylation predicts temozolomide benefit; trial-eligible patients have superior outcomes.

• No survival advantage was observed for proton versus photon therapy in adjusted analyses.

**Supplementary Information:**

The online version contains supplementary material available at 10.1007/s11060-026-05572-w.

## Introduction

Glioblastoma (WHO 2021 grade 4), defined by IDH-wildtype status and associated molecular features, remains a uniformly fatal diagnosis despite maximal therapy, with median survival ranging from 8 to 20 months [1–3]. Prognostic factors such as O6-methylguanine-DNA methyltransferase (MGMT) promoter methylation, extent of resection, and patient age are well-established; however, most survival models are often derived from clinical trial populations that may not reflect real-world treatment variability [2–12]. Radiation-induced lymphopenia (RIL) has also emerged as a negative prognostic factor across multiple cancer types, including glioblastoma [13–16], yet its independent association with survival in molecularly defined glioblastoma treated with standard chemoradiotherapy remains incompletely defined. Real-world data analyzing the survival outcomes and prognostic factors in this population are limited, particularly in cohorts receiving modern radiotherapy (RT) techniques, temozolomide (TMZ), and molecular profiling. Moreover, real-world studies frequently report survival outcomes inferior to those observed in clinical trials, highlighting the need for contemporary, population-specific prognostic models.

In this retrospective study, the largest single-institutional analysis to date, we evaluated clinical, molecular, treatment-related, and hematologic predictors of survival in a large, single-institution cohort of adults with glioblastoma as defined by WHO 2021 treated with maximal safe resection and adjuvant RT. Our objective was to clarify independent prognostic factors, including RIL, in the context of a comprehensive survival model reflective of contemporary glioma care.

## Methods

### Patient cohort

We identified consecutive patients *≥* 18 years old with a diagnosis of high-grade glioblastoma at a single institution from January 2014-December 2024. Inclusion criteria were: (1) histologic diagnosis of adult-type diffuse glioma; (2) absence of an IDH-mutation confirmed by immunohistochemistry (IHC) or next-generation sequencing (NGS); (3) histologic features consistent with grade 4 glioma (including necrosis and microvascular proliferation) or molecular diagnosis of glioblastoma per WHO 2021 criteria (i.e., IDH-wt with TERT promoter mutation, EGFR amplification, or Chr7 gain/Chr10 loss); and (4) receipt of adjuvant RT following maximal safe resection. Patients with pediatric-type high-grade gliomas, diffuse midline glioma (H3 K27–altered), H3 G34–mutant tumors, IDH-mutant infiltrating gliomas, or gliosarcoma were excluded (Supplementary Fig. 1).

Due to changes in diagnostic workflows over the study period, IDH status was determined by IHC, PCR, or NGS. Patients with unknown IDH status (e.g., pathology results missing) were marked as “unknown” but were included if they met histologic or molecular criteria for glioblastoma per WHO 2021 guidelines, although these constituted only a small portion of the cohort (0.2%) [17, 18]. MGMT status was assessed via qualitative methylation-specific PCR across CpG sites of the MGMT promoter region. Patients were classified as “low” or “high” positive MGMT if below or above 10% methylated across the promoter region per institutional guidelines; any degree of methylation was considered MGMT-methylated for this study.

### Identification of clinical trial eligible cohort

A “clinical trial–eligible” subset of patients was also defined using key eligibility criteria from the Stupp trials protocol, including age ≤ 70 years, ECOG performance status ≤ 2, baseline absolute neutrophil count ≥ 1.5 × 10⁹/L, and platelet count ≥ 100 × 10⁹/L [2, 3].

### Surgical resection, radiotherapy, and systemic treatment

All patients underwent maximal safe resection or biopsy, with extent of resection (gross total [GTR] or subtotal [STR]) determined by postoperative magnetic resonance imaging (MRI), wherein GTR was defined as removal of all contrast enhancing tumor per Response Assessment in Neuro-Oncology (RANO) criteria [19]. Within six to eight weeks of resection, patients initiated adjuvant RT, delivered as either conventionally fractionated (60 Gy in 30 fractions) or hypofractionated (40.05 Gy in 15 fractions) regimens, as determined by the treating radiation oncologist based on patient age, performance status, and comorbidities [20, 21].

Conventionally fractionated RT planning followed institutional and NRG guidelines with two dose levels: 60 Gy delivered to the resection cavity and residual contrast-enhancing gross tumor volume (GTV), and 46 Gy to regions of T2/FLAIR hyperintensity plus a 1.5–2.0 cm margin to define the clinical target volume (CTV), constrained to anatomic boundaries [22]. Hypofractionated RT was delivered to a CTV consisting of the resection cavity, any residual contrast-enhancing GTV, and T2/FLAIR signal plus a 1.5 cm margin cropped to anatomic boundaries. RT was delivered using either photons or protons. For proton therapy, pencil beam scanning (PBS) or passive scatter techniques were used. Patients receiving a mix of proton and photon therapy were categorized by the modality comprising the majority of fractions. Concurrent and/or adjuvant temozolomide (TMZ) was offered the discretion of the treating clinician. Starting in 2017, tumor treating fields (TTFields) were offered starting four weeks post-RT for eligible patients.

### Labratory biomarkers

Complete blood counts (CBCs) were extracted from 30 days before RT start to 120 days after RT completion. Collected parameters included white blood cell (WBC) count, hemoglobin (HGB), platelets (PLT), absolute neutrophil count (ANC), and absolute lymphocyte count (ALC). Baseline labs were calculated as the average of all available values within 30 days prior to RT initiation. ALC and ANC nadirs were defined as the lowest value recorded from RT start to four months post-RT.

Lymphopenia was graded per CTCAE v5.0 [23]: grade 0 (ALC > 1.0 × 10⁹/L), grade 1 (1.0–0.8 × 10⁹/L), grade 2 (0.8–0.5 × 10⁹/L), grade 3 (0.5–0.2 × 10⁹/L), and grade 4 (< 0.2 × 10⁹/L). Severe radiation-induced lymphopenia (sRIL) was defined as grade ≥ 3 lymphopenia consistent with prior analyses of radiation-induced lymphopenia in glioma and other solid tumors [13, 24–26]. As the majority of patients received concurrent TMZ, which is independently lymphotoxic, sRIL in this cohort likely reflects the combined immunosuppressive effects of chemoradiotherapy rather than radiation alone.

### Endpoints and statistical analysis

The primary objective was to identify clinical, molecular, tumor-related, and hematologic factors associated with overall survival (OS), with particular emphasis on the prognostic impact of sRIL. Secondary objectives included evaluating the prognostic performance of these factors in a trial-eligible subgroup and assessing the association between radiotherapy modality and survival outcomes. OS was measured from the start date of adjuvant RT until death; patients lost to follow up or alive at last encounter were censored.

Descriptive statistics were reported as medians with interquartile ranges (IQR) or frequencies with percentages, with differences between groups calculated by Wilcoxon rank sum test, Pearson’s Chi-squared test, or Fisher’s exact test where appropriate. Time-to-event endpoints were evaluated with the Kaplan-Meier method and groups were compared with the log-rank test. Median follow-up time was calculated using the reverse Kaplan–Meier method. Univariable and multivariable Cox proportional hazards models were used to assess predictors of OS. For variable selection in multivariable modeling, we applied the least absolute shrinkage and selection operator (LASSO) method, a penalized regression technique that improves model interpretability and reduces overfitting. This approach was particularly suited to address multicollinearity among laboratory biomarkers (e.g., pre-/post-RT ALC, ANC, and WBC). The lambda tuning parameter of LASSO method was selected to minimize the prediction error. Proportional hazards assumptions were tested using global and individual Schoenfield residuals and covariates violating the assumption were modeled using stratification to allow baseline hazards to vary across strata. Analyses evaluating predictive TMZ benefit were restricted to concurrent TMZ exposure and did not include adjuvant TMZ to mitigate immortal-time bias. We also evaluated the relationship between ALC nadir, age, and OS using non-parametric natural cubic spline regression due to non-linearity, as demonstrated in likelihood ratio tests.

Missing data for variables including pre-/post-RT laboratory values (e.g., ALC, HGB, WBC, and ANC, missing in approximately 5–45% of patients) were addressed using multiple imputation using chained equations (MICE). Missing data patterns were reviewed and found to be consistent with a missing-at-random (MAR) mechanism, based on structured block missingness patterns related to laboratory panel ordering and the absence of a clinically meaningful association between missingness and key prognostic variables. Notably, the primary outcome variable of interest, post-RT ALC, used to calculate sRIL, was only missing in 12.7% of patients. Variables with more than 50% missingness were excluded from the imputation model. Predictive mean matching (PMM) was used for continuous and categorical predictors, except for the key outcome variable post-RT ALC nadir, which exhibited a non-linear relationship with other covariates and was therefore imputed using classification and regression trees (CART). The predictor matrix was customized to prioritize pre-RT lymphocyte values as predictors of ALC nadir. Five imputations with ten iterations each were performed to ensure convergence, and imputed data were assessed using strip and density plots. Post-imputation diagnostics including strip plots and density plots were evaluated to confirm the plausibility of the imputed values. As a sensitivity analysis, the primary multivariable model was repeated using m = 20 imputations and in a complete-case cohort restricted to patients with observed values for all covariates (*N* = 394). All statistical tests were two-sided; p-value of < 0.05 was considered significant. Analyses were conducted in R version 4.2.2 (R Foundation, Vienna, Austria) using the glmnet package for LASSO and rms v6.6.0 for spline regression.

## Results

### Patient cohort

We identified 832 patients with a diagnosis of glioblastoma that met inclusion criteria. Details of those patients are shown in Table 1; median age was 64 years (IQR 57–71), 61% were male, and 77% were Caucasian. MGMT promoter methylation was absent in 54%, and the majority had glioblastoma confirmed by both histologic and molecular criteria (64%). GTR was achieved in 33%, STR in 59%, and biopsy alone in 8%. Conventionally fractionated radiotherapy was delivered in 73% of patients with 57% receiving photon therapy. Differences in the patient cohort by receipt of proton therapy are shown in Supplementary Table 1. Concurrent TMZ was administered in 94%, and 69% received adjuvant TMZ, with a median of 5 cycles (IQR 3–6). TTFields were used in 11%. Median pre-RT ALC and ANC were 1.40 × 10⁹/L and 5.8 × 10⁹/L, respectively; median nadir post-RT ALC was 0.60 × 10⁹/L, and ANC was 3.06 × 10⁹/L. After RT, lymphopenia severity was distributed as follows: grade 0 in 155 patients (19%), grade 1 in 142 (17%), grade 2 in 270 (32%), grade 3 in 215 (26%), and grade 4 in 50 (6%); 265 (32%) had sRIL.

**Table 1:** Baseline patient and tumor characteristics

Variable	*n* = 832^1^
Age (years)	64 (57–71)
Gender	
Male	508 (61)
Female	324 (39)
Race	
Caucasian	642 (77)
African American	50 (6.0)
Asian	21 (2.5)
Other	119 (14)
Marital Status	
Not Partnered	210 (25)
Partnered	593 (71)
Unknown	29 (3.5)
BMI (kg/m²)*	26.8 (23.8–29.9)
IDH Status	
Wild Type	830 (99.8)
Unknown	2 (0.2)
MGMT Status	
Methylated	331 (40)
Unmethylated	450 (54)
Unknown	51 (6.1)
GBM Definition	
Histologic only	207 (25)
Molecular only	88 (11)
Both	535 (64)
Extent of Resection	
GTR	271 (33)
STR	491 (59)
Biopsy	65 (7.8)
Unknown	5 (0.6)
Pre-RT ECOG*	
0	113 (16)
1	342 (42)
2	217 (27)
3	126 (15)
Adjuvant RT Regimen	
Conventional	604 (73)
Hypofractioned	228 (27)
RT Modality	
Photon	477 (57)
Proton	355 (43)
Any TMZ	787 (95)
Concurrent TMZ with RT	783 (94)
Adjuvant TMZ after RT	571 (69)
Adjuvant TMZ cycles	5 (3–6)
Used TTF	93 (11)
Pre-RT WBC (K/uL)	8.5 (6.4–11.6)
Pre-RT HGB (g/dL)	12.9 (11.8–13.8)
Pre-RT PLT (x10^9^ cells/L)	242 (198–308)
Pre-RT ALC (x10^9^ cells/L)	1.40 (1.00–1.91)
Pre-RT ANC (x10^9^ cells/L)	5.8 (4.3–8.9)
Pre-RT NLR	4 (3–8)
Post-RT WBC nadir (K/uL)	4.50 (3.42–6.10)
Post-RT HGB nadir (g/dL)	12.50 (11.20–13.60)
Post-RT PLT nadir (x10^9^ cells/L)	152 (109–193)
Post-RT ALC nadir (x10^9^ cells/L)	0.60 (0.40–0.90)
Post-RT ANC nadir (x10^9^ cells/L)	3.06 (2.00–4.49)

^1^Median (IQR) or Frequency (%)

Abbreviations: IQR = interquartile range; BMI = body mass index; IDH = isocitrate dehydrogenase; MGMT = O6-methylguanine-DNA methyltransferase; GBM = glioblastoma multiforme; NOS = not otherwise specified; GTR = gross total resection; STR = subtotal resection; RT = radiotherapy; TMZ = temozolomide; TTF = tumor treating fields; WBC = white blood cell; HGB = hemoglobin; PLT = platelet; ALC = absolute lymphocyte count; ANC = absolute neutrophil count; NLR = neutrophil to lymphocyte ratio

*N is less than 832 due to missing records

### Survival outcomes

**Fig. 1:**
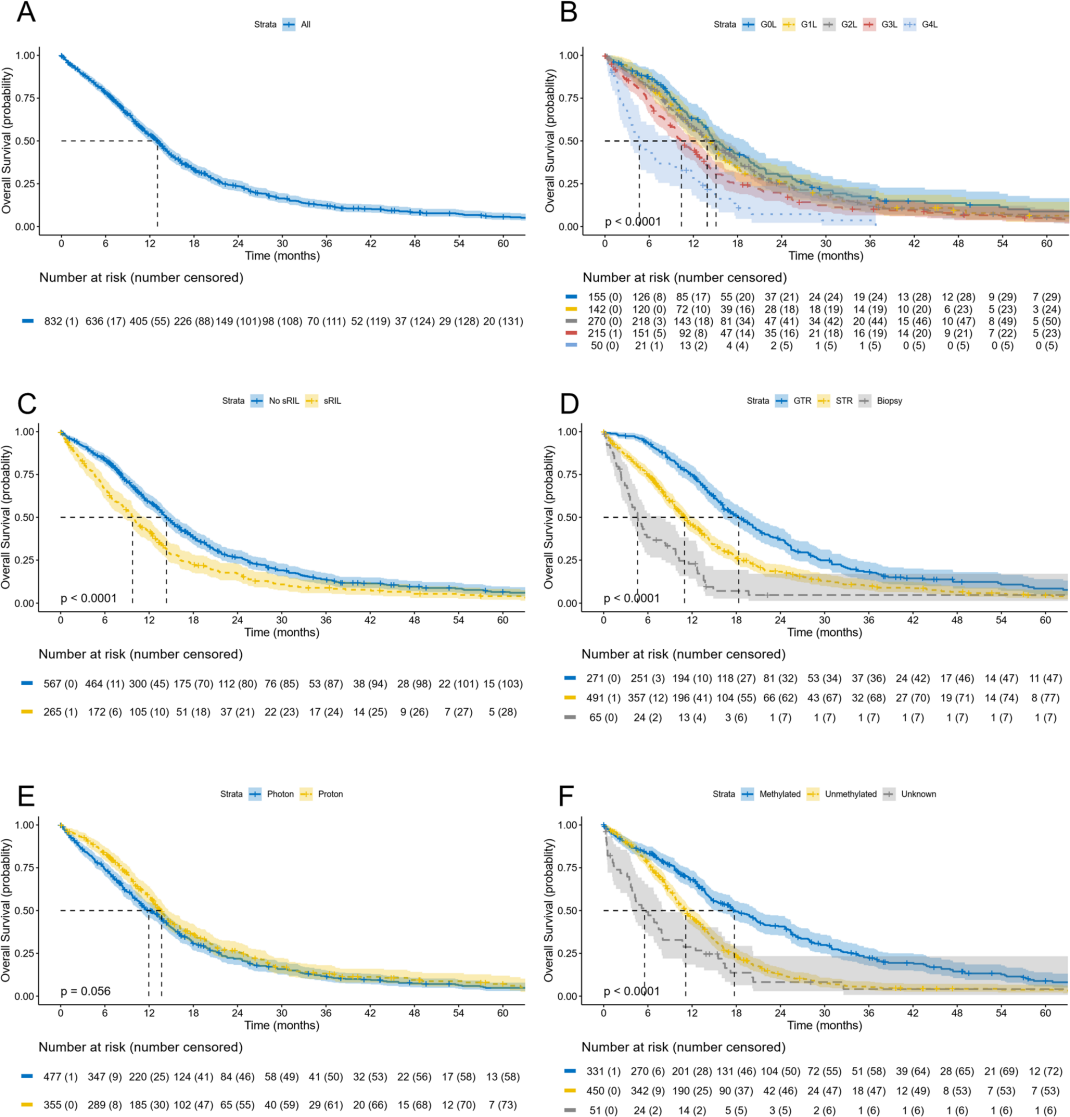
Kaplan–Meier curves for overall survival (OS) outcomes for the cohort. Panel **A** shows the OS for all patients. Panel **B** displays OS stratified by increasing grade of lymphopenia, per CTCAE v5. Panel **C** stratifies OS by the presence of severe radiation-induced lymphopenia (sRIL; grade 3 + lymphopenia). Panel **D** displays OS by the extent of maximal safe resection, where GTR = gross total resection and STR = subtotal resection. Panel E shows survival by the receipt of proton or photon radiotherapy. Panel **F** displays OS by O6-methylguanine-DNA methyltransferase (MGMT) status. Tick marks represent censored events, and the dashed line represents the median value. Shaded areas represent 95% confidence intervals. P-values are from the log-rank test

Median follow up time was 50 months (IQR 26–81); there were 687 deaths with a median OS of 13.0 months (95% CI 12.1–13.8) (Fig. 1A). On unadjusted Kaplan-Meier analysis worse OS was observed in patients with increasing grade of lymphopenia (median 15.1 vs. 13.9 vs. 13.9vs 10.4 vs. 4.7 months, *p* < 0.001) (Fig. 1B), sRIL (median 9.8 vs. 14.4 months, *p* < 0.001) (Fig. 1C), biopsy or STR vs. GTR (median 4.6 vs. 11.0 vs. 18.3 months, *p* < 0.001) (Fig. 1D), receipt of photon therapy (median 12.0 vs. 13.7 months, *p* = 0.056) (Fig. 1E), and MGMT unmethylated tumors (median 12.6 vs. 19.1 months, *p* < 0.001) (Fig. 1F). Conventional RT (median 14.9 vs. 7.7 months, *p* < 0.001) and use of TTF (median 18.5 vs. 12.5 months, *p* < 0.001) were also associated with better OS.

Univariable Cox regression modeling is shown in Supplementary Table 2. On multivariable modeling (Table 2), OS was associated with increasing age (HR 1.02, 95% CI 1.01-1.0, *p* < 0.001), MGMT unmethylated tumors (HR 2.19, 95% CI 1.84–2.61, *p* < 0.001), STR (HR 1.42, 95% CI 1.19–1.69, *p* < 0.001) or biopsy (HR 2.77, 95% CI 1.99–3.85, *p* < 0.001), use of concurrent TMZ (HR 0.72, 95% CI 0.53–0.99, *p* = 0.047), use of TTF (HR 0.67, 95% CI 0.52–0.87, *p* = 0.003), post-RT ANC nadir (HR 1.15, 95% CI 1.11–1.18, *p* < 0.001), pre-RT neutrophil nadir (HR 1.09, 95% CI 1.01–1.17, *p* = 0.02), and sRIL(HR 1.37, 95% CI 1.16–1.63, *p* < 0.001). The use of proton therapy was associated with a non-significant but favorable estimate of OS across the confidence interval (HR 0.87, 95% CI 0.74–1.03, *p* = 0.11). In a stratified Cox proportional hazards model adjusting for race, resection status, MGMT methylation, ECOG, and sRIL, we evaluated the effect of radiation modality, laboratory values, and age on OS (Supplementary Table 3). Age (HR 1.03, 95% CI 1.02–1.04, *p* < 0.001) and post-RT ANC nadir (HR 1.16, 95% CI 1.11–1.21, *p* < 0.001) was associated with inferior survival. In the stratified model, proton therapy was not associated with a significant difference in OS compared to photon therapy (HR 0.90, 95% CI 0.74–1.09, *p* = 0.3). A 4-month landmark analysis accounting for guarantee-time in the ascertainment of sRIL and TTFields initiation is presented in Supplementary Table 4; HR estimates for key variables were significant and directionally consistent with the primary model. In sensitivity analyses, the primary imputed model was repeated using m = 20 imputations and in a complete-case cohort. The sRIL hazard ratio was identical between the m = 5 primary model and the m = 20 sensitivity analysis (HR 1.37, 95% CI 1.15–1.62, *p* < 0.001), confirming stability of the imputation approach. In the complete-case cohort (*N* = 394), sRIL remained directionally consistent with the primary model (HR 1.24, 95% CI 0.96–1.60), with wider confidence intervals reflecting the reduced sample size.

**Table 2:** Multivariable cox regression analysis for overall survival

Variable	HR (95% CI)	*P*-value
Age (continuous, per year)	**1.02 (1.01–1.03)**	**< 0.001**
Race (ref: white)		
Black	0.71 (0.50–1.02)	0.061
Asian	1.36 (0.80–2.30)	0.3
Other	1.24 (0.98–1.56)	0.074
MGMT Status (ref: Methylated)		
Unmethylated	**2.19 (1.84–2.61)**	**< 0.001**
Unknown	**2.86 (2.01–4.05)**	**< 0.001**
Extent of Resection (ref: GTR)		
STR	**1.42 (1.19–1.69)**	**< 0.001**
Biopsy	**2.77 (1.99–3.85)**	**< 0.001**
Pre-RT ECOG (ref: 0)		
One	1.02 (0.81–1.29)	0.8
Two	**1.68 (1.30–2.16)**	**< 0.001**
Three	**2.71 (2.04–3.61)**	**< 0.001**
RT Modality (ref: Photon)		
Proton	0.87 (0.74–1.03)	< 0.11
Concurrent TMZ (ref: No)		
Yes	**0.72 (0.53–0.99)**	**0.047**
Used TTF (ref: No)		
Yes	**0.67 (0.52–0.87)**	**0.003**
Pre-RT WBC (per 1 K/uL)	1.02 (1.00-1.04)	0.06
Pre-RT HGB (per 1 g/dL)	0.98 (0.93–1.03)	0.4
Post-RT ANC nadir (per 0.1 × 10^9^ cells/L)	**1.15 (1.11–1.18)**	**< 0.001**
Pre-RT Neutrophil nadir (per 0.1 × 10^9^ cells/L)	**1.09 (1.01–1.17)**	**0.02**
sRIL (ref: no)		
Yes	**1.37 (1.16–1.63)**	**< 0.001**

Abbreviations: Ref = reference group; MGMT = O6-methylguanine-DNA methyltransferase; GTR = gross total resection; STR = subtotal resection; RT = radiotherapy; TMZ = temozolomide; sRIL = severe radiation-induced lymphopenia (grade 3 + lymphopenia). Bolded values are significant (*p* < 0.05)

**Fig. 2:**
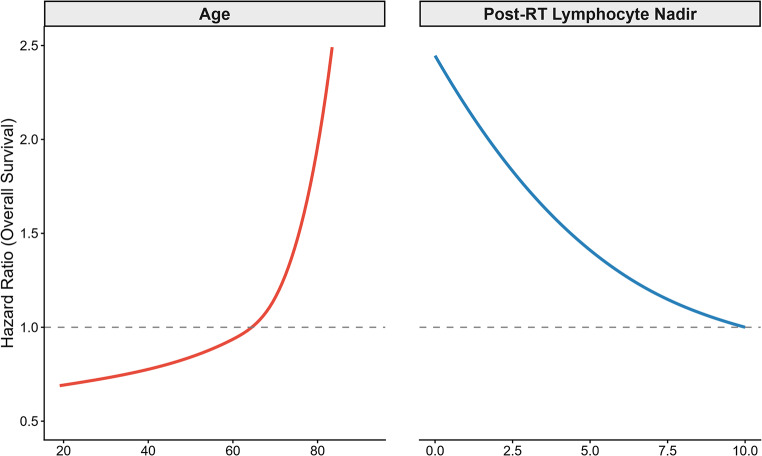
Non-linear associations between overall survival (OS) and (**A**) age and (**B**) post-radiotherapy absolute lymphocyte count (ALC) nadir, modeled using restricted cubic splines. Hazard ratios (HRs) were estimated from multivariable Cox models including spline terms, with all other covariates held at their median values. HR = 1 corresponds to the reference risk at the median of each variable

The relationship between OS, age, and lymphocyte counts were significantly non-linear. A restricted cubic spline Cox model provided superior fit compared to a linear model (likelihood ratio test: χ²=17.1, df = 4, *p* = 0.002) and thus were modeled with non-parametric cubic spline regression (Fig. 2). This model demonstrated that both age at treatment and post-radiotherapy ALC nadir demonstrated significant non-linear associations with OS. The hazard of death increased with older age, most steeply beyond age 60–65. Lower post-RT lymphocyte nadir was associated with an elevated hazard of death, with the steepest increase in risk at the lowest ALC values below approximately 0.5-1.0 × 10⁹ cells/L.

### MGMT as predictive biomarker and clinical trial eligle subset

In the overall cohort, we evaluated MGMT promoter methylation as a predictive biomarker for benefit from concurrent TMZ using an interaction model. In a multivariable Cox proportional hazards model adjusting for age, sex, extent of resection, ECOG, and fractionation scheme, a significant interaction was observed between MGMT status and concurrent TMZ use (p_interaction_ <0.001), indicating effect modification by MGMT promoter methylation. Among patients with MGMT-methylated tumors, receipt of concurrent TMZ was associated with improved OS (HR 0.10, 95% CI 0.04–0.29, *p* < 0.001). In contrast, among patients with MGMT-unmethylated tumors, concurrent TMZ was not significantly associated with OS (HR 0.78, 95% CI 0.55–1.10, *p* = 0.16). Consistent with these findings, the magnitude of TMZ-associated benefit differed substantially by MGMT status, with a marked survival advantage confined to patients with MGMT-methylated tumors (Supplementary Fig. 2).

**Fig. 3:**
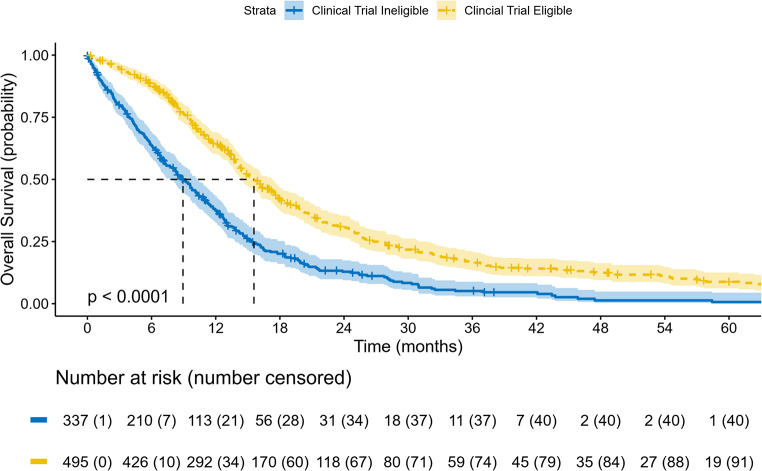
Overall survival (OS) for patients stratified by clinical trial eligibility. Tick marks represent censored events, and the dashed line represents the median value. The p-value is calculated from the log-rank test for Kaplan-Meier curves

Among the 832 patients, 495 (59.5%) met clinical trial eligibility criteria, according to Stupp protocol. Clinical trial eligible patients had significantly longer OS compared to ineligible patients (median OS 15.6 vs. 8.9 months, *p* < 0.001; Fig. 3). In this population, there was no statistically significant difference in OS between patients that received proton and photon therapy (median OS 16.9 vs. 15.3 months, *p* = 0.14). Clinical trial eligibility was associated with improved OS within both MGMT promoter methylation subgroups (Supplementary Fig. 3). Trial-eligible patients had significantly longer median OS than trial-ineligible patients among those with MGMT-methylated tumors (24.6 vs. 12.4 months) and MGMT-unmethylated tumors (13.7 vs. 7.4 months; both *p* < 0.001). Survival among trial-eligible patients with MGMT-unmethylated tumors approximated that of trial-ineligible patients with MGMT-methylated tumors.

## Discussion

In the largest, single-institution, real-world cohort of patients with glioblastoma treated with RT in the modern molecular era, we identified multiple clinical, molecular, and treatment-related variables associated with OS, including MGMT promoter methylation, extent of resection, use of concurrent TMZ, post-RT lymphopenia, and neutrophil nadir. Notably, we demonstrate that sRIL remains a strong negative prognostic factor even after adjusting for key clinical and molecular variables. Importantly, our findings confirm that contemporary real-world survival outcomes in the molecular era mirror those of major clinical trials [2, 4, 27], and underscore the prognostic relevance of treatment-associated immunologic changes and motivate prospective investigation of immune-sparing approaches in glioma management. Recent prognostic efforts in glioblastoma have increasingly leveraged high-dimensional imaging and molecular features, including radiomics, radiogenomics, methylomic signatures, and machine learning–based subgrouping, to refine risk stratification and survival prediction [28–30]. These approaches have demonstrated that intratumoral heterogeneity and integrated clinical–omic models provide additional prognostic resolution beyond traditional clinicopathologic variables. However, many of these models rely on specialized data streams that are not routinely available in real-world practice.

Prognostic markers in glioblastoma have been studied in both trial and real-world settings [4–8, 11, 12, 31–35]. Consistent with prior literature, we observed improved OS with younger age, GTR, MGMT methylation, and receipt of standard chemoradiotherapy. These prognostic factors are similar to previous identified in secondary analyses of global, randomized trials and published nomograms [12]. While prior studies have linked lymphopenia with inferior outcomes across several cancer types [25, 26, 36, 37] and in glioblastoma specifically [13–16], few if any studies have integrated these immunological markers into a multivariable survival model that includes molecular markers and maximal treatment. Here, we demonstrated that sRIL was consistently associated with worse survival, supporting its evaluation as a prognostic biomarker for clinical risk stratification. This novel and robust OS model provides one of the most comprehensive assessments of prognostic factors in a modern cohort to date.

There are multiple potential mechanisms to explain the association between sRIL and survival. First, sRIL may reflect a composite of tumor-driven immune suppression and treatment toxicity. Cranial irradiation, especially when targeting large target volumes or midline structures, exposes circulating lymphocytes to lymphotoxic doses, potentially impairing the anti-tumor immune response [38, 39]. Additionally, that the independent association of elevated post-RT neutrophil counts with worse OS, is consistent with prior observations of immunologic imbalance characterized by lymphocyte depletion and pro-inflammatory myeloid dominance, though causal inference is not possible from these data [40, 41].

Second, is that non-random exposures (e.g., steroid and TMZ use) influence lymphocyte counts resulting in residual confounding and misattribution of effect. TMZ is well known to be lymphotoxic, with dose-dependent and prolonged effects on CD4 + T-cell populations and has previously been associated with treatment-related lymphopenia [42]. The observed sRIL rate of 32% is consistent with rates reported in prospective chemoradiotherapy cohorts, where concurrent TMZ has been associated with rates of severe lymphopenia approximately three times higher than those observed with radiotherapy alone, underscoring the contribution of TMZ to treatment-associated immunosuppression in this population [21]. Additionally, long-term steroid use results in lymphocyte depletion and redistribution and can blunt T-cell mediated immunity. Patients requiring high-dose steroids, often those with bulky disease and edema, incomplete resection, or neurologic symptoms, are also those with poorer prognosis. Thus, lower ALC values post-RT may reflect not only radiation toxicity but also steroid exposure and disease severity. Importantly, the intent of this study was not to determine the causal etiology of this association, and further mechanistic studies are needed to definitively prove the causal link between sRIL and poor oncologic outcomes. While we were unable to fully adjust for steroid use in this analysis, sRIL remained independently associated with survival after multivariable adjustment, though residual confounding cannot be excluded.

Although proton therapy was associated with non-significantly but numerically improved OS in adjusted Kaplan-Meier analysis and a favorable HR in the initial multivariable model, this association was not maintained in stratified analyses accounting for non-proportional hazards. It was also not replicated in a cohort of clinical trial eligible patients. We elected not to perform propensity score matching, as this approach would likely not resolve confounding by indication [43]. Retrospective comparative effective analysis is not the appropriate avenue for proving the survival benefit of proton therapy. The observed association may reflect patient selection bias, as those chosen for proton therapy were likely more robust and medically fit. This is supported by the early and sustained separation of survival curves in unadjusted analysis (Fig. 1E). While the survival benefit of proton therapy remains unproven, protons may offer advantages in reducing treatment-related toxicity and lymphopenia by limiting low-dose exposure to circulating lymphocytes and hematopoietic tissues [24, 44–46]. Preliminary results from the NRG BN001 phase II randomized trial suggest a potential OS benefit with dose-intensified proton therapy in GBM, and this hypothesis is currently being evaluated in an ongoing phase III study [47]. Prospective randomized trials are needed to validate these hypotheses.

Our findings also contextualize the predictive role of MGMT promoter methylation within a real-world population characterized by substantial heterogeneity in treatment eligibility. Trial-ineligible patients experienced inferior OS irrespective of MGMT status, underscoring the strong prognostic influence of baseline clinical fitness. Nearly 40% of patients in our cohort would not have met eligibility criteria for landmark chemoradiotherapy trials [2, 3]. These ineligible patients experienced inferior OS, irrespective of MGMT status, underscoring the strong prognostic influence of baseline clinical fitness. In the overall cohort, the observed interaction between MGMT promoter methylation and concurrent TMZ was consistent with the established predictive biology of MGMT methylation, with a survival advantage associated with TMZ use confined to patients with MGMT-methylated tumors (Supplementary Fig. 2). However, we caution that this analysis is limited by the small and likely non-random comparator group – only 6% of patients did not receive concurrent TMZ – and patients who did not receive TMZ likely reflect confounding by indication, including poor performance status or clinical frailty, rather than a planned treatment omission. These findings should therefore be interpreted as hypothesis-generating and consistent with prior randomized evidence rather than as a new demonstration of predictive effect in an observational dataset. Nonetheless, recent trials such as CheckMate 498 [48] and NRG BN007 [49] have demonstrated inferior outcomes in patients with MGMT-unmethylated GBM treated without TMZ, suggesting that omission of TMZ may be detrimental even in this subgroup. The observed detriment was not attributed to immunotherapy toxicity but rather to the absence of alkylating chemotherapy, solidifying the use of TMZ in unmethylated GBM as standard of care. Together, these findings suggest that while clinical trial eligibility identifies a prognostically favorable subset of patients, MGMT promoter methylation continues to function as a clinically relevant biomarker for TMZ responsiveness. Moreover, survival among trial-eligible patients with MGMT-unmethylated tumors approximated that of trial-ineligible patients with MGMT-methylated disease, highlighting the dominant prognostic weight of molecular subtype relative to treatment intensification alone.

Our analysis has several limitations. As a retrospective cohort, unmeasured variables—such as detailed molecular alterations or resected tumor volume [50]–may have influenced outcomes. Our cohort includes only patients who received radiotherapy, potentially excluding those with poor performance status or rapidly progressive disease. We did not capture the use, dose, or duration of corticosteroids, which may confound hematologic outcomes. Additionally, we were unable to assess progression-free survival and did not record second-line therapies or enrollment on a clinical trial at our institution, which can influence post-progression survival. Finally, long-term immune recovery after RT was not evaluated.

## Conclusion

This study highlights the importance of integrating clinical, molecular, and immunologic variables when modeling survival in IDH-wt HGG. Severe lymphopenia remains a significant and independent prognostic factor, supporting the investigation for lymphocyte-sparing strategies and closer immune monitoring. Further prospective studies are warranted to investigate whether interventions such as proton therapy or immunologic support are associated with reduced immunosuppression and improve outcomes in this patient population.

## Supplementary Information

Below is the link to the electronic supplementary material.

Supplementary Material 1

Supplementary Material 2

Supplementary Material 3

Supplementary Material 4

Supplementary Material 5

Supplementary Material 6

Supplementary Material 7

Supplementary Material 8

## Data Availability

Data is institutionally protected but may be made available upon reasonable request.
