# Supplementary figures and images for: Clinical, molecular, and immunologic determinants of survival in WHO-defined IDH-wildtype glioblastoma treated with radiotherapy: a large real-world cohort study
Source: J Neurooncol. 2026 Apr 25;177(3):125. doi: 10.1007/s11060-026-05572-w (PMC13110210; doi:10.1007/s11060-026-05572-w)

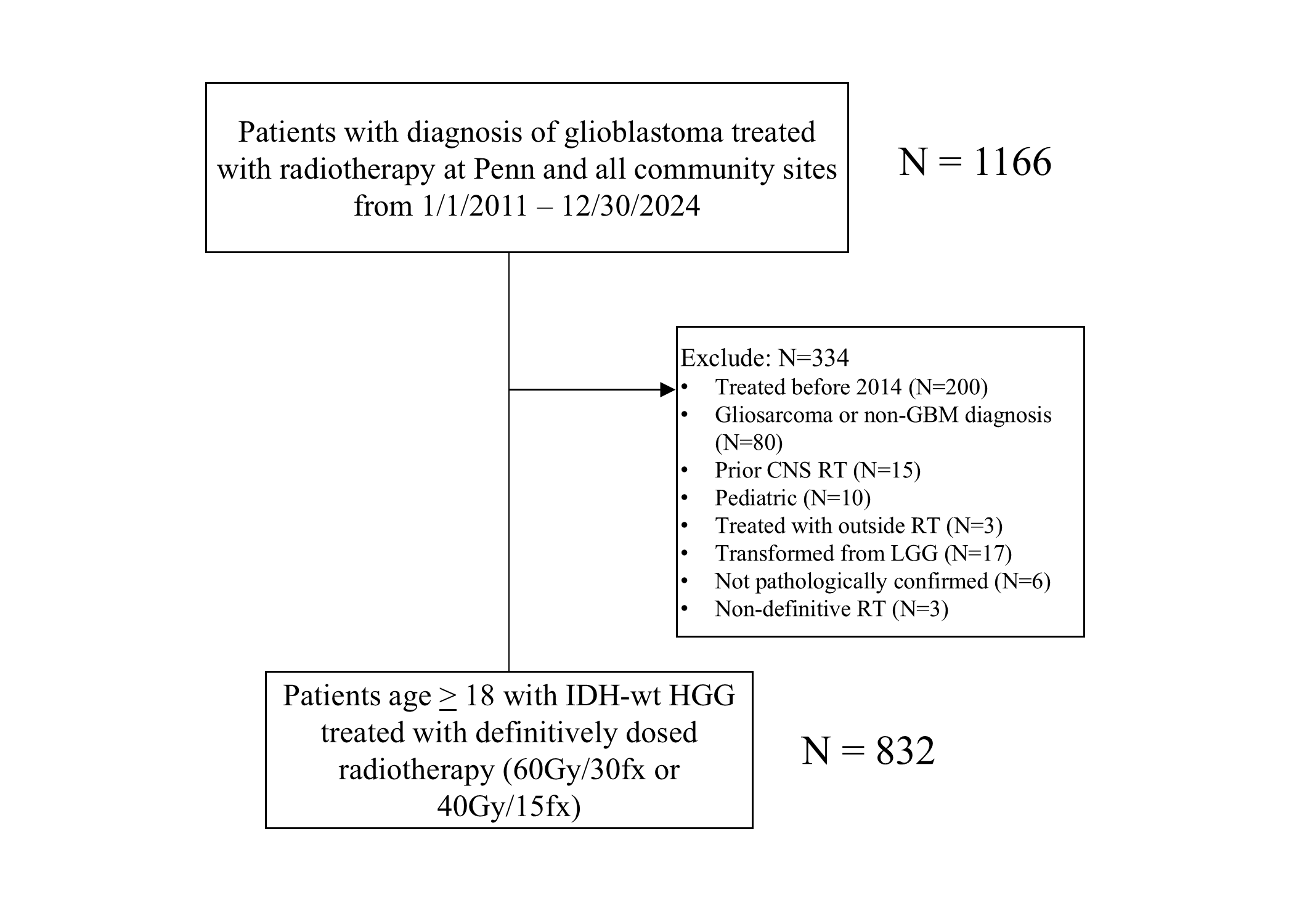

Supplement: Supplementary file 1 — Supplementary Material 1 [file 11060_2026_5572_MOESM1_ESM.png]

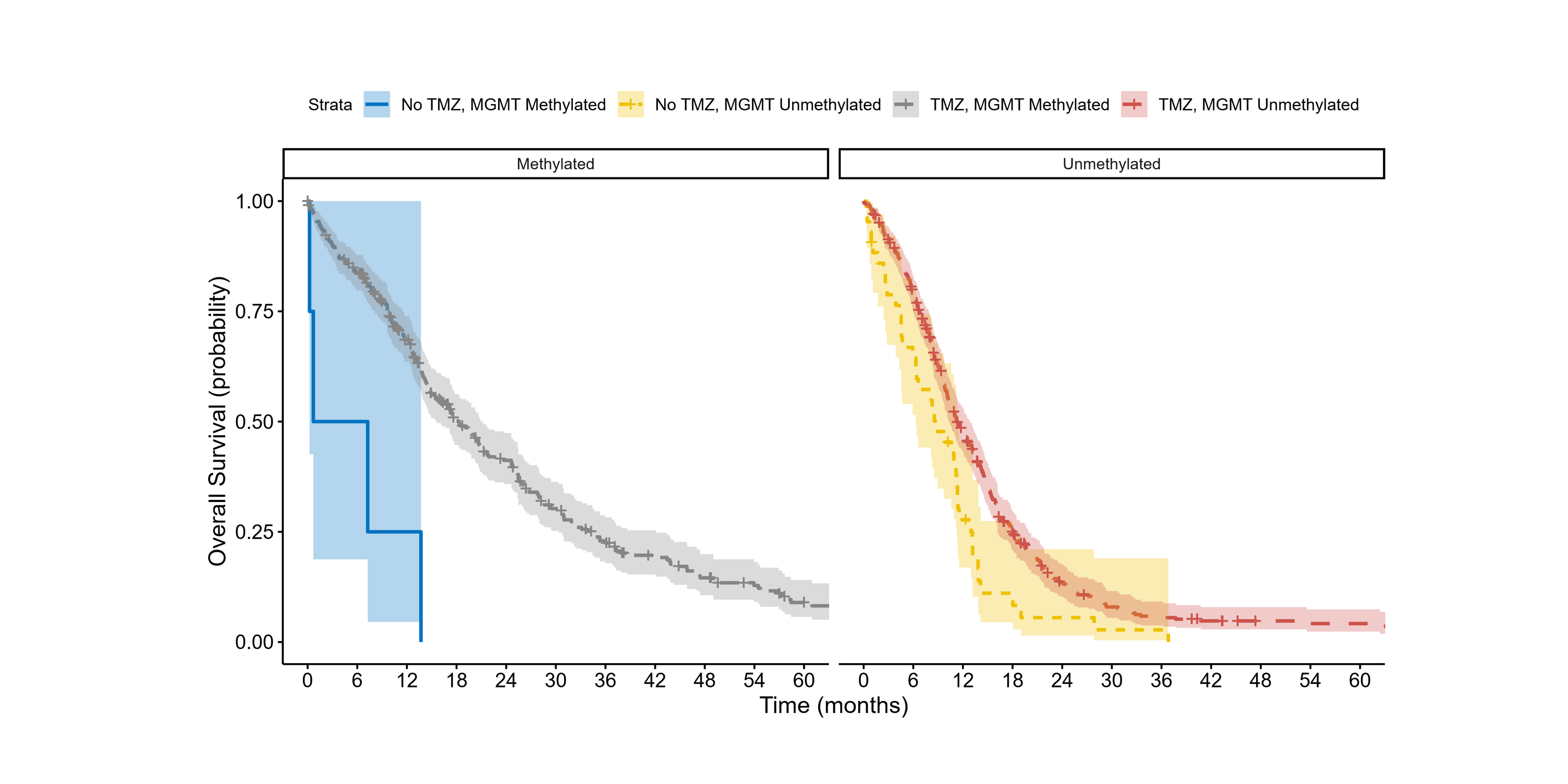

Supplement: Supplementary file 2 — Supplementary Material 2 [file 11060_2026_5572_MOESM2_ESM.png]

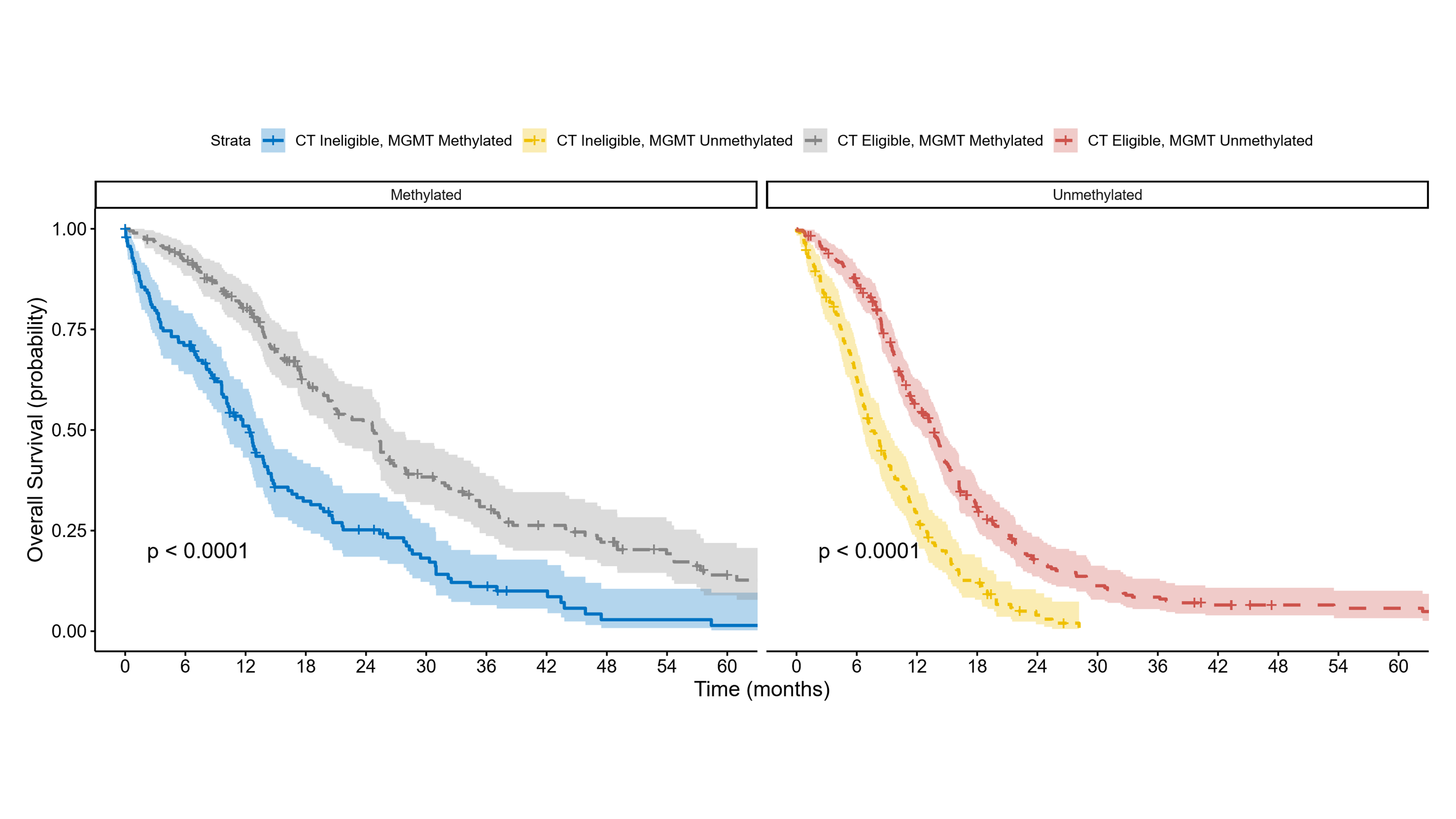

Supplement: Supplementary file 3 — Supplementary Material 3 [file 11060_2026_5572_MOESM3_ESM.png]
