# Supplementary material for: Clinical, molecular, and immunologic determinants of survival in WHO-defined IDH-wildtype glioblastoma treated with radiotherapy: a large real-world cohort study
Source: J Neurooncol. 2026 Apr 25;177(3):125. doi: 10.1007/s11060-026-05572-w (PMC13110210; doi:10.1007/s11060-026-05572-w)
Supplement: Supplementary file 4 — Supplementary Material 4 [file 11060_2026_5572_MOESM4_ESM.docx]

Supplemental Table 1. Difference in the study cohort stratified by the receipt of proton or photon therapy.

| **Variable** | **Overall**  N = 832^1^ | **Photon**  N = 477^1^ | **Proton**  N = 355^1^ | **p-value**^2^ |
| --- | --- | --- | --- | --- |
| **Age** | 64 (57 - 71) | 63 (56 - 70) | 66 (58 - 72) | **0.003** |
| **Sex** |  |  |  | **0.001** |
| Male | 508 (61) | 314 (66) | 194 (55) |  |
| Female | 324 (39) | 163 (34) | 161 (45) |  |
| **Race** |  |  |  | **<0.001** |
| White | 642 (77) | 355 (74) | 287 (81) |  |
| Black | 50 (6.0) | 22 (4.6) | 28 (7.9) |  |
| Asian | 21 (2.5) | 12 (2.5) | 9 (2.5) |  |
| Other | 119 (14) | 88 (18) | 31 (8.7) |  |
| **BMI** | 26.8 (23.8 - 29.9) | 26.6 (23.8 - 29.8) | 26.9 (23.8 - 30.2) | 0.33 |
| **Marital Status** |  |  |  | **0.014** |
| Not Partnered | 210 (25) | 123 (26) | 87 (25) |  |
| Partnered | 593 (71) | 330 (69) | 263 (74) |  |
| Unknown | 29 (3.5) | 24 (5.0) | 5 (1.4) |  |
| **IDH Status** |  |  |  | 0.51 |
| Wild Type | 830 (100) | 475 (100) | 355 (100) |  |
| Unknown | 2 (0.2) | 2 (0.4) | 0 (0) |  |
| **MGMT Status** |  |  |  | **0.001** |
| Methylated | 331 (40) | 176 (37) | 155 (44) |  |
| Unmethylated | 450 (54) | 260 (55) | 190 (54) |  |
| Unknown | 51 (6.1) | 41 (8.6) | 10 (2.8) |  |
| **Resection Status** |  |  |  | 0.070 |
| GTR | 271 (33) | 139 (29) | 132 (37) |  |
| STR | 491 (59) | 293 (61) | 198 (56) |  |
| Unknown | 5 (0.6) | 4 (0.8) | 1 (0.3) |  |
| Biopsy | 65 (7.8) | 41 (8.6) | 24 (6.8) |  |
| **Adjuvant RT Regimen** |  |  |  | 0.67 |
| Conventional | 604 (73) | 349 (73) | 255 (72) |  |
| Hypofractionated | 228 (27) | 128 (27) | 100 (28) |  |
| **Any TMZ** | 787 (95) | 448 (94) | 339 (95) | 0.32 |
| **Concurrent TMZ with RT** | 783 (94) | 445 (93) | 338 (95) | 0.24 |
| **Adjuvant RMZ after RT** | 571 (69) | 314 (66) | 257 (72) | **0.043** |
| **Adjuvant TMZ cycles** | 5.0 (3.0 - 6.0) | 6.0 (3.0 - 9.0) | 5.0 (2.0 - 6.0) | **<0.001** |
| **Used TTF** | 93 (11) | 54 (11) | 39 (11) | 0.88 |
| **Pre-RT WBC (K/uL)** | 8.5 (6.4 - 11.6) | 8.4 (6.3 - 11.3) | 8.6 (6.4 - 11.6) | 0.65 |
| **Pre-RT HGB (g/dL)** | 12.90 (11.80 - 13.80) | 13.10 (11.90 - 13.90) | 12.80 (11.70 - 13.70) | 0.053 |
| **Pre-RT PLT (x10^9^ cells/L)** | 242 (198 - 308) | 245 (202 - 310) | 236 (187 - 299) | 0.092 |
| **Pre-RT PLT (x10^9^ cells/L)** | 5.8 (4.3 - 8.9) | 5.9 (4.2 - 9.1) | 5.8 (4.4 - 8.8) | 0.82 |
| **Pre-RT ALC (x10^9^ cells/L)** | 1.40 (1.00 - 1.91) | 1.40 (1.00 - 1.90) | 1.50 (1.03 - 1.97) | 0.086 |
| **Post-RT WBC nadir (K/uL)** | 4.50 (3.42 - 6.10) | 4.50 (3.40 - 6.10) | 4.60 (3.50 - 6.00) | 0.89 |
| **Post-RT HGB nadir (g/dL)** | 12.50 (11.20 - 13.60) | 12.70 (11.30 - 13.70) | 12.40 (11.10 - 13.50) | 0.072 |
| **Post-RT PLT nadir (x10^9^ cells/L)** | 152 (109 - 193) | 150 (109 - 191) | 154 (107 - 197) | 0.49 |
| **Post-RT ALC nadir (x10^9^ cells/L)** | 0.60 (0.40 - 0.90) | 0.60 (0.40 - 0.90) | 0.60 (0.40 - 0.90) | 0.97 |
| **Post-RT ANC nadir (x10^9^ cells/L)** | 3.06 (2.00 - 4.49) | 3.10 (2.00 - 4.70) | 3.00 (2.00 - 4.30) | 0.50 |
| **Grade of Post-RT Lymphopenia** |  |  |  | 0.77 |
| Grade 0 | 155 (19) | 93 (19) | 62 (17) |  |
| Grade 1 | 142 (17) | 75 (16) | 67 (19) |  |
| Grade 2 | 270 (32) | 154 (32) | 116 (33) |  |
| Grade 3 | 215 (26) | 126 (26) | 89 (25) |  |
| Grade 4 | 50 (6.0) | 29 (6.1) | 21 (5.9) |  |
| **sRIL** | 265 (32) | 155 (32) | 110 (31) | 0.64 |
| ^1^Median (IQR) or Frequency (%) | | | | |
| ^2^Wilcoxon rank sum test; Pearson's Chi-squared test; Fisher's exact test | | | | |

Abbreviations: IQR = interquartile range; BMI = body mass index; IDH = isocitrate dehydrogenase; MGMT = O6-methylguanine-DNA methyltransferase; GBM = glioblastoma multiforme; GTR = gross total resection; STR = subtotal resection; RT = radiotherapy; TMZ = temozolomide; TTF = tumor treating fields; WBC = white blood cell; HGB = hemoglobin; PLT = platelet; ALC = absolute lymphocyte count; ANC = absolute neutrophil count; NLR = neutrophil to lymphocyte ratio.
