# Supplementary material for: Clinical, molecular, and immunologic determinants of survival in WHO-defined IDH-wildtype glioblastoma treated with radiotherapy: a large real-world cohort study
Source: J Neurooncol. 2026 Apr 25;177(3):125. doi: 10.1007/s11060-026-05572-w (PMC13110210; doi:10.1007/s11060-026-05572-w)
Supplement: Supplementary file 5 — Supplementary Material 5 [file 11060_2026_5572_MOESM5_ESM.docx]

Supplemental Table 2. Univariable Cox Regression Analysis of Overall Survival.

| **Characteristic** | **HR** | **95% CI** | **p-value** |
| --- | --- | --- | --- |
| Age | 1.02 | 1.02, 1.03 | <0.001 |
| Race |  |  |  |
| White | — | — |  |
| Black | 0.76 | 0.54, 1.06 | 0.10 |
| Asian | 0.94 | 0.56, 1.57 | 0.8 |
| Other | 1.35 | 1.09, 1.67 | 0.007 |
| Sex |  |  |  |
| Male | — | — |  |
| Female | 0.99 | 0.85, 1.16 | >0.9 |
| BMI | 1.00 | 1.00, 1.00 | 0.7 |
| Marital Status |  |  |  |
| Not Partnered | — | — |  |
| Partnered | 0.90 | 0.76, 1.07 | 0.2 |
| Unknown | 1.79 | 1.17, 2.74 | 0.008 |
| MGMT Status |  |  |  |
| Methylated | — | — |  |
| Unmethylated | 1.85 | 1.57, 2.17 | <0.001 |
| Unknown | 2.77 | 2.01, 3.83 | <0.001 |
| GBM Definition |  |  |  |
| Histologic | — | — |  |
| Molecular | 0.67 | 0.50, 0.89 | 0.006 |
| Both | 0.88 | 0.74, 1.04 | 0.13 |
| Unknown | 4.45 | 1.10, 18.0 | 0.036 |
| Resection Status |  |  |  |
| GTR | — | — |  |
| STR | 1.70 | 1.44, 2.00 | <0.001 |
| Unknown | 1.87 | 0.77, 4.55 | 0.2 |
| Biopsy | 3.86 | 2.87, 5.20 | <0.001 |
| Pre-RT ECOG |  |  |  |
| 0 | -- | -- | -- |
| 1 | 1.03 | 0.82, 1.29 | 0.8 |
| 2 | 1.56 | 1.23, 1.99 | **<0.001** |
| 3 | 2.77 | 2.12, 3.62 | **<0.001** |
| Adjuvant RT Regimen |  |  |  |
| Conventional | — | — |  |
| Hypofractionated | 2.16 | 1.83, 2.56 | <0.001 |
| RT Modality |  |  |  |
| Photon | — | — |  |
| Proton | 0.86 | 0.74, 1.00 | 0.057 |
| Any TMZ |  |  |  |
| No | — | — |  |
| Yes | 0.43 | 0.32, 0.59 | <0.001 |
| Concurrent TMZ with RT |  |  |  |
| No | — | — |  |
| Yes | 0.44 | 0.33, 0.60 | <0.001 |
| Adjuvant TMZ after RT |  |  |  |
| No | — | — |  |
| Yes | 0.37 | 0.32, 0.44 | <0.001 |
| Adjuvant TMZ cycles | 0.86 | 0.83, 0.88 | <0.001 |
| Used TTF |  |  |  |
| No | — | — |  |
| Yes | 0.61 | 0.48, 0.79 | <0.001 |
| Pre-RT WBC (K/uL) | 1.02 | 1.01, 1.04 | 0.007 |
| Pre-RT HGB (g/dL) | 0.98 | 0.93, 1.03 | 0.5 |
| Pre-RT PLT (x10^9^ cells/L) | 1.00 | 1.00, 1.00 | 0.3 |
| Pre-RT ALC (x10^9^ cells/L) | 1.00 | 1.00, 1.00 | >0.9 |
| Pre-RT ANC (x10^9^ cells/L) | 1.03 | 1.01, 1.05 | <0.001 |
| Post-RT WBC nadir (K/uL) | 1.11 | 1.08, 1.15 | <0.001 |
| Post-RT HGB nadir (g/dL) | 0.95 | 0.91, 0.99 | 0.010 |
| Post-RT PLT nadir (x10^9^ cells/L) | 1.00 | 1.00, 1.00 | 0.12 |
| Post-RT ALC nadir (x10^9^ cells/L) | 0.63 | 0.51, 0.77 | <0.001 |
| Post-RT ANC nadir (x10^9^ cells/L) | 1.16 | 1.12, 1.19 | <0.001 |
| sRIL |  |  |  |
| No | — | — |  |
| Yes | 1.16 | 1.12, 1.19 | <0.001 |
| Grade of Lymphopenia |  |  |  |
| Grade 0 | — | — |  |
| Grade 1 | 1.16 | 0.90, 1.49 | 0.3 |
| Grade 2 | 1.16 | 0.93, 1.45 | 0.2 |
| Grade 3 | 1.50 | 1.19, 1.89 | <0.001 |
| Grade 4 | 2.79 | 1.98, 3.95 | <0.001 |
