# Supplementary material for: Clinical, molecular, and immunologic determinants of survival in WHO-defined IDH-wildtype glioblastoma treated with radiotherapy: a large real-world cohort study
Source: J Neurooncol. 2026 Apr 25;177(3):125. doi: 10.1007/s11060-026-05572-w (PMC13110210; doi:10.1007/s11060-026-05572-w)
Supplement: Supplementary file 6 — Supplementary Material 6 [file 11060_2026_5572_MOESM6_ESM.docx]

Supplemental Table 3. Stratified Multivariable Cox Regression Model for Overall Survival.

| Variable | HR (95% CI) | P-value |
| --- | --- | --- |
| Age (continuous, per year) | **1.03 (1.02-1.04)** | **<0.001** |
| RT Modality (ref: Photon) |  |  |
| Proton | 0.90 (0.740-1.09) | 0.3 |
| Concurrent TMZ (ref: No) |  |  |
| Yes | 0.85 (0.58-1.24) | 0.4 |
| Used TTF (ref: No) |  |  |
| Yes | 0.83 (0.61-1.12) | 0.2 |
| Pre-RT WBC (per 1 K/uL) | 1.02 (1.00-1.04) | 0.07 |
| Pre-RT HGB (per 1 g/dL) | 0.94 (0.91-1.04) | 0.4 |
| Post-RT ANC nadir per (0.1 x 10^9^ cells/L) | **1.16 (1.11-1.21)** | **<0.001** |
| Post-RT Neutrophil nadir per (0.1 x 10^9^ cells/L) | 0.98 (0.52-1.83) | 0.9 |

Abbreviations: Ref = reference group; MGMT = O6-methylguanine-DNA methyltransferase; GTR = gross total resection; STR = subtotal resection; RT = radiotherapy; TMZ = temozolomide; sRIL = severe radiation-induced lymphopenia (grade 3+ lymphopenia). Bolded values are significant (p<0.05).
