# Supplementary material for: Clinical, molecular, and immunologic determinants of survival in WHO-defined IDH-wildtype glioblastoma treated with radiotherapy: a large real-world cohort study
Source: J Neurooncol. 2026 Apr 25;177(3):125. doi: 10.1007/s11060-026-05572-w (PMC13110210; doi:10.1007/s11060-026-05572-w)
Supplement: Supplementary file 7 — Supplementary Material 7 [file 11060_2026_5572_MOESM7_ESM.docx]

Supplementary Table 4. Landmark Analysis at 4 Months in Primary Multivariable Cox Regression Model

| **Characteristic** | **N** | **Event N** | **HR** | **95% CI** | **p-value** |
| --- | --- | --- | --- | --- | --- |
| **Age** | 688 | 556 | **1.02** | **1.01, 1.03** | **<0.001** |
| **Race** |  |  |  |  |  |
| *White* | 546 | 449 | — | — |  |
| *Black* | 42 | 31 | 0.69 | 0.47, 1.01 | 0.059 |
| *Asian* | 19 | 13 | 1.41 | 0.80, 2.49 | 0.2 |
| *Other* | 81 | 63 | 0.95 | 0.71, 1.26 | 0.7 |
| **MGMT Status** |  |  |  |  |  |
| *Methylated* | 278 | 201 | — | — |  |
| *Unmethylated* | 380 | 330 | **2.61** | **2.15, 3.16** | **<0.001** |
| *Unknown* | 30 | 25 | **2.73** | **1.74, 4.30** | **<0.001** |
| **Extent of Resection** |  |  |  |  |  |
| *GTR* | 262 | 209 | — | — |  |
| *STR* | 390 | 317 | **1.34** | **1.12, 1.62** | **0.002** |
| *Biopsy* | 32 | 26 | **2.61** | **1.69, 4.02** | **<0.001** |
| **Pre-RT ECOG** |  |  |  |  |  |
| *Zero* | 127 | 99 | — | — |  |
| *One* | 314 | 242 | 1.00 | 0.79, 1.27 | >0.9 |
| *Two* | 174 | 147 | **1.41** | **1.08, 1.85** | **0.012** |
| *Three* | 73 | 68 | **2.31** | **1.67, 3.20** | **<0.001** |
| **RT Modality** |  |  |  |  |  |
| *Photon* | 380 | 321 | — | — |  |
| *Proton* | 308 | 235 | 0.95 | 0.79, 1.13 | 0.5 |
| **Concurrent TMZ** |  |  |  |  |  |
| *No* | 34 | 32 | — | — |  |
| *Yes* | 654 | 524 | 0.75 | 0.51, 1.10 | 0.14 |
| **Used TTF** |  |  |  |  |  |
| *No* | 597 | 492 | — | — |  |
| *Yes* | 91 | 64 | **0.74** | **0.57, 0.97** | **0.031** |
| **Pre-RT WBC (per 1 K/uL)** | 688 | 556 | **1.02** | **1.00, 1.05** | **0.028** |
| **Pre-RT HGB (per 1 g/dL)** | 688 | 556 | 1.00 | 0.94, 1.06 | >0.9 |
| **Post-RT ANC nadir (per 0.1 x 10^9^ cells/L)** | 688 | 556 | **1.16** | **1.11, 1.21** | **<0.001** |
| **Pre-RT Neutrophil nadir (per 0.1 x 10^9^ cells/L)** | 688 | 556 | 1.08 | 1.00, 1.17 | 0.063 |
| **sRIL** |  |  |  |  |  |
| *No* | 492 | 386 | — | — |  |
| *Yes* | 196 | 170 | **1.36** | **1.12, 1.64** | **0.002** |
| Abbreviations: CI = Confidence Interval, HR = Hazard Ratio | | | | | |
