# Supplementary material for: Clinical, molecular, and immunologic determinants of survival in WHO-defined IDH-wildtype glioblastoma treated with radiotherapy: a large real-world cohort study
Source: J Neurooncol. 2026 Apr 25;177(3):125. doi: 10.1007/s11060-026-05572-w (PMC13110210; doi:10.1007/s11060-026-05572-w)
Supplement: Supplementary file 8 — Supplementary Material 8 [file 11060_2026_5572_MOESM8_ESM.docx]

Supplementary Figure 1. CONSORT diagram depicting the identification of the final cohort.

Supplementary Figure 2. Kaplan–Meier curves for overall survival (OS) stratified by receipt of temozolomide (TMZ) and faceted by O6-methylguanine–DNA methyltransferase (MGMT) promoter methylation status. Tick marks represent censored events, and the dashed line represents the median value. Shaded areas represent 95% confidence intervals. P-values are from the log-rank test.

Supplementary Figure 3. Overall survival (OS) for patients stratified by clinical trial eligibility and faceted by O6-methylguanine-DNA methyltransferase (MGMT) methylation status. Tick marks represent censored events, and the dashed line represents the median value. Shaded areas represent 95% confidence intervals. P-values are from the log-rank test.
